# Acceptability and satisfaction towards self‐collection for chlamydia and gonorrhoea testing among transgender women in Tangerine Clinic, Thailand: shifting towards the new normal

**DOI:** 10.1002/jia2.25801

**Published:** 2021-09-08

**Authors:** Akarin Hiransuthikul, Rena Janamnuaysook, Linrada Himma, Chiraporn Taya, Tidarat Amatsombat, Phongdanai Chumnanwet, Kritima Samitpol, Artsanee Chancham, Jiratchaya Kongkapan, Jeeranuch Rueannak, Pintusorn Getwongsa, Peevara Srimanus, Nipat Teeratakulpisarn, Narukjaporn Thammajaruk, Matthew Avery, Tanyaporn Wansom, Stephen Mills, Reshmie A. Ramautarsing, Nittaya Phanuphak

**Affiliations:** ^1^ Institute of HIV Research and Innovation (IHRI) Bangkok Thailand; ^2^ Department of Preventive and Social Medicine, Faculty of Medicine Chulalongkorn University Bangkok Thailand; ^3^ Centre of Excellence in Transgend Health (CETH) Chulalongkorn University Bangkok Thailand; ^4^ FHI 360 and LINKAGES Bangkok Thailand

**Keywords:** acceptability, chlamydia, gonorrhea, satisfaction, self‐collection, transgender women

## Abstract

**Introduction:**

Provider‐collected swabs are an unappealing procedure for many transgender women and may have led to suboptimal rates of *Chlamydia trachomatis* (CT) and *Neisseria gonorrhoeae* (NG) testing. Self‐collection for CT/NG testing is recommended for men who have sex with men. However, the information on acceptability and clinical performance to support a recommendation for transgender women is lacking. We aimed to determine the acceptability and satisfaction towards self‐collection for CT/NG testing among Thai transgender women.

**Methods:**

Thai transgender women who attended Tangerine Clinic (a transgender‐led, integrated, gender‐affirming care and sexual health services clinic in Bangkok, Thailand) between May and July 2020 and had condomless sexual intercourse within the past six months were offered to collect urine and perform self‐swabs of pharyngeal, rectal, and if applicable, neovaginal compartments for pooled nucleic acid amplification testing for CT/NG infections. Participants received a diagram, video and oral instructions about how to perform self‐collection procedure. Those who accepted self‐collection were also offered to receive provider collection to evaluate the performance between the two methods. Self‐administered questionnaires were used to assess satisfaction.

**Results:**

Among 216 transgender women enrolled, 142 (65.7%) accepted self‐collection. All who accepted had pharyngeal, rectal and urine samples collected. Of 31 transgender women who had undergone genital surgery, 28 (90.3%) accepted neovaginal self‐swab. The acceptance rate increased from 46.2% in May to 84.5% in July 2020. One participant had an invalid result. All transgender women who accepted self‐collection could perform it without assistance, and 82.8% were highly satisfied with the method. None reported dissatisfaction. Due to the COVID‐19 pandemic, provider collection services were discontinued early, and only eight transgender women were able to perform both methods for performance evaluation. The performance agreement was 100%.

**Conclusions:**

Thai transgender women had high acceptability and satisfaction towards self‐collection for CT/NG testing. The performance was promising compared to provider collection. Our results support the implementation of self‐collection to the sexually transmitted infection services, particularly during the COVID‐19 pandemic where physical distancing is the new normal. A larger study is warranted to determine the performance of self‐collection for CT/NG testing in each anatomical compartment and confirm the performance between self‐collection and provider collection.

## 1 INTRODUCTION

*Chlamydia trachomatis* (CT) and *Neisseria gonorrhoeae* (NG) infections are serious global health issues and disproportionately affect key populations, including transgender women [1, 2]. Globally, the overall prevalence of CT/NG infections among transgender women ranged variedly between regions and studies, and can be as high as 28.6% for CT infection and 21.4% for NG infection [3, 4]; a substantially higher number compared to the general population (2.7% to 4.2% for CT infection and 0.6% to 0.8% for NG infection) [1, 2]. Data from community‐based clinics using provider‐collected swabs among sexually active Thai transgender throughout Thailand found that CT and NG infection prevalence was 23.0% and 14.5% women, respectively [5]. High prevalence of CT/NG infections is a concern because of their association with HIV acquisition and transmission [1, 2]. Unfortunately, the lack of routine screening may result in a missed opportunity to diagnose and treat these curable infections, as most CT/NG infections are asymptomatic.

Even if screening was made, samplings from an inadequate number of anatomical sites could still lead to missed diagnoses, particularly among transgender women [3, 5]. In a previous study conducted by our group using provider‐collected swabs, 46% to 69% of CT/NG infections among transgender women were isolated from a single anatomical site. If urine screening was conducted alone, a 94% of CT/NG cases among transgender women would have been missed [5]. While there are currently no separate guidelines for transgender women, the US Centers for Disease Control and Prevention (CDC) recommends that healthcare providers assess all possible anatomy and patterns of sexual behaviour among transgender women [6]. However, the traditional provider‐collected swabs might be unappealing to transgender individuals. Concerns over discrimination and insensitivity in healthcare settings, lack of trained healthcare providers, perceived provider incompetence, gender dysphoria centred around the genitals or cultural inappropriateness of showing their bodies to the other gender can pose barriers to CT/NG screening [7, 8, 9, 10].

To avoid such issues, self‐collection, which is a private and simple noninvasive procedure, could be an alternative to provider‐collected sampling and provide an opportunity to enhance active case finding. In addition, this may also be beneficial and encouraging for transgender women clients to enter sexually transmitted infection (STI) screening services during the COVID‐19 pandemic when physical distancing is recommended. Our primary objective was to determine the acceptability and satisfaction towards self‐collection for CT/NG testing among Thai transgender women attending Tangerine Clinic, a transgender‐led, integrated, gender‐affirming care and sexual health service in Bangkok, Thailand.

## 2 METHODS

### 2.1 Enrolment of participants

Tangerine Clinic was established in 2015 and has served 3989 transgender women through March 2021. Tangerine Clinic is Asia's first sexual health clinic for transgender people, based in Bangkok, Thailand. The clinic is managed by trained transgender staff and gender‐sensitive medical professionals and offers comprehensive health services such as sexual health, HIV and STI testing, gender‐affirming hormone treatment and referrals for gender‐affirming surgery and legal assistance. Most services are free of charge, including STI testing and treatment. Approximately 75% of all transgender clients are reached through the social media influencers. The majority came for gender‐affirming hormone treatment, and over 90% came for HIV services.

All transgender women who reported condomless sexual intercourse within the past six months were routinely offered CT/NG testing from all anatomical sites (i.e., pharyngeal swabs, rectal swabs, urine and, if applicable, neovaginal swabs) regardless of their self‐reported exposure site(s) using self‐collected methods between May and July 2020. Those who accepted self‐collection were also offered to receive provider collection to evaluate the performance between the two methods.

Self‐collection was initially planned as an option for those who do not prefer provider collection starting May 2020. However, provider collection was discontinued early in the same month in response to the growing impact of the COVID‐19 pandemic to promote physical distancing. Consequently, those who refused self‐collection were unable to test for CT/NG infections using provider collection. Moreover, those who accepted self‐collection were no longer offered to receive provider collection for performance evaluation. Self‐administered questionnaires were used to assess participants’ demographic data and risk behaviours. An additional questionnaire was used to assess participants’ satisfaction towards self‐collection among those who accepted self‐collection.

### 2.2 Specimen collection and diagnostic tests

The staff gave the clients a diagram and a video instruction (Figure 1, a video instruction in Thai can be found in Supporting file S1 and at https://www.youtube.com/watch?v=hqg‐725AmEA) and also oral instructions about how to perform self‐collection. Except for participants who were enrolled in the performance evaluation between self‐collection and provider collection, samples were pooled and tested for CT/NG infections using nucleic acid amplification testing (NAAT; Abbott Real Time CT/NG, Abbott Molecular Inc., IL, USA). Participants who tested positive for CT/NG infection received treatment according to the World Health Organization's (WHO) recommendations [11, 12].

**Figure 1:**
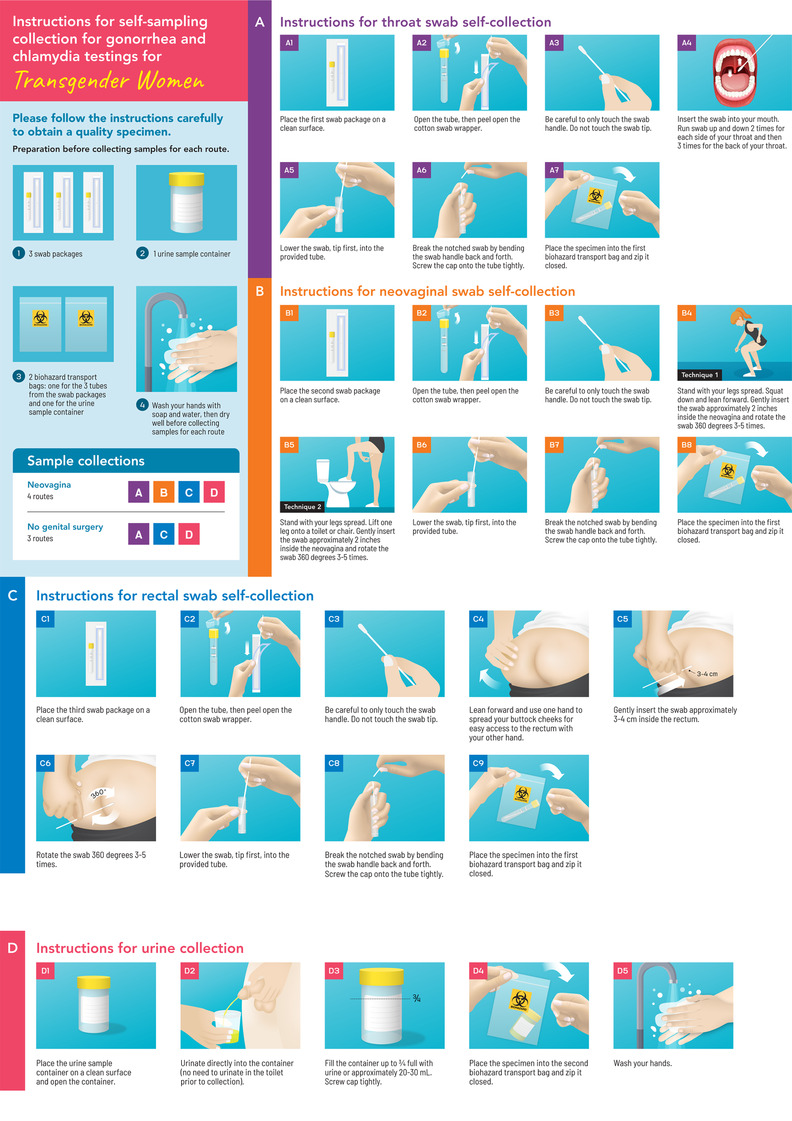
Illustrated instructions for self‐sampling collection for gonorrhea and chlamydia testing in English.

### 2.3 Ethical approval

Tangerine Clinic data collection and analysis were approved by the Institutional Review Board of the Faculty of Medicine, Chulalongkorn University, Bangkok, Thailand (IRB No. 158/56). All clients provided verbal consent before receiving services at Tangerine Clinic.

### 2.4 Statistical analysis

Demographic data, risk behaviours and responses to self‐collection were summarized as median (interquartile range (IQR)) and number (percentage) for continuous and categorical variables, respectively. Characteristics were compared between those who accepted and refused self‐collection Mann‐Whitney *U*‐test and a chi‐square or Fisher's exact test as appropriate. Multivariate logistic regression based on covariates associated with outcomes in univariate regression with *p*‐value of <0.15 was used to identify associated with accepting self‐collection. Statistical significance was defined as *p*‐value of <0.05. Data in variables with more than 20% missing were excluded from the analysis. Analysis was performed using Stata 15 (StataCorp, College Station, TX).

## 3 RESULTS

Of a total of 216 transgender women who were offered to perform self‐collection, 142 (65.7%) accepted. Besides a modest but significantly older age among transgender women accepted self‐collection, and there were no other significant differences in characteristics between those who accepted and refused self‐collection (Table 1). The self‐collection acceptance rate gradually increased from 46.2% in May to 84.5% in July (Table 2).

**Table 1:** Characteristics of 216 transgender women according to self‐collection acceptance who attended Tangerine Clinic, Bangkok, Thailand between May and July 2020

	**Accepted self‐collection**		**Refused self‐collection**		
	**(*n* = 142)**		**(*n* = 74)**		
Characteristics	**n**	***%***	**n**	***%***	p‐Value
Median age (IQR) years	27.9		26.7		0.03^*^
	(25.2 to 31.5)		(22.9 to 28.6)		
Highest education					0.95
Secondary school or less	39/116	33.6	23/66	34.8	
Vocational study	18/116	15.5	11/66	16.7	
Bachelor degree or higher	59/116	50.9	32/66	48.5	
Main occupation					0.50
Unemployed	10/130	7.7	5/69	7.2	
Student	13/130	10.0	11/69	15.9	
Employed, non‐sex work	48/130	36.9	28/69	40.6	
Employed, sex work	59/130	45.4	25/69	36.2	
Income^a^					0.93
<20,000 THB/month	52/101	51.5	33/65	50.8	
≥20,000 THB/month	49/101	48.5	32/65	49.2	
Marital status: single	140/142	98.6	73/74	98.7	0.69
Number of sexual partners in the past six months					0.26
Single partner	108/141	76.6	62/72	86.1	
Multiple partners	6/141	4.3	2/72	2.8	
Refuse to answer	27/141	19.1	8/72	11.1	
Group sex in the past six months	3/141	2.1	0/73	0.0	0.55
Illicit drug use in the past six months	2/141	1.4	0/73	0.0	0.55
HIV infection^b^	1/59	1.7	0/64	0.0	0.48

Abbreviation: THB, Thai Baht.

^a^ 20,000 THB = approximately 612 USD (conversion rate 32.7 THB = 1 USD).

^b^ All were tested at the study visit.

* *p*‐Value of <0.05.

**Table 2:** Acceptance rate of 216 transgender women who attended Tangerine Clinic, Bangkok, Thailand between May and July 2020

**Period**	**No. of clients offered**	**No. of clients who accepted self‐collection**	**Self‐collection acceptance rate (%)**
May	78	36	46.2
June	80	57	71.3
July	58	49	84.5
Total	216	142	65.7

Among 142 transgender women who accepted self‐collection, all had samples collected from pharyngeal swabs, rectal swabs and urine; and 28 out of 31 (90.3%) transgender women who underwent genital surgery had an additional sample collected from neovaginal swabs. Results from self‐collection of all but one transgender woman (99.3%) were valid; the only one invalid result was due to faecal contamination from rectal swabs. The prevalence of CT and NG infections among transgender women performing self‐collection was 22.5% (32/142) and 17.6% (25/142), respectively.

All transgender women who accepted self‐collection could perform it without assistance, and 82.8% were highly satisfied with the method (Table 3). None reported dissatisfaction. Overall, 69.5% found self‐collection to be simple, and 65.6% expressed no concern with this sample collection method. The most common concern over self‐collection was that improper technique might lead to incorrect results (33.6%). Up to 97% would choose to do self‐collection again in the future, and all would recommend this method to their peers. Using multivariable logistic regression, we found no characteristic that was significantly associated with accepting self‐collection (Supporting file S2).

**Table 3:** Self‐collection questionnaire among transgender women who accepted self‐collection

**Items**	***n***	**%**
Self‐perceived risk for STI acquisition		
No risk	8/128	6.3
Low risk	46/128	35.9
Moderate risk	56/128	43.8
High risk	18/128	14.1
First time for self‐collection	109/128	85.2
Feeling towards self‐collection		
Simple process	89/128	69.5
Neither too simple nor difficult	1/128	0.8
Difficult	38/128	29.7
Most concern over self‐collection		
No concern	84/128	65.6
Improper technique might lead to incorrect results	43/128	33.6
Unfavourable place to perform self‐collection	1/128	0.8
Will choose to do self‐collection again in the future	124/128	96.9
Want to have home‐based self‐collection in the future	88/128	68.8
Will recommend self‐collection to peers	128/128	100
Overall satisfaction with self‐collection		
Dissatisfied	0/128	0
Neutral	22/128	17.2
Highly satisfied	106/128	82.8

Abbreviation: STI, sexually transmitted infections.

Due to the COVID‐19 pandemic during the study period, provider collection services were discontinued early, and only eight transgender women were able to do both self‐collection and provider collection for performance evaluation. The performance agreement was 100%. All had samples collected from pharyngeal swabs, rectal swabs and urine; and six transgender women had an additional sample from neovaginal swabs. The performance agreement between self‐collection and provider collection was 100%.

## 4 DISCUSSION

We demonstrated high acceptability of self‐collection for CT/NG infections among Thai transgender women, with an 89% increase in acceptance rate during the three‐month period of launching the service. Transgender women also showed high satisfaction towards self‐collection, and only one‐third expressed any concern about the method, mainly regarding the correct technique of performing self‐collection.

Increased uptake of CT/NG testing is among the first step to strengthen the continuum of STI prevention, diagnosis, treatment and care [13]. However, an STI testing rate of as low as 18% was demonstrated among sexually active transgender women in the United States despite having high‐risk behaviours [14]. Multiple factors such as underlying anxiety or feeling of uneasiness towards providers and past experiences of discrimination in healthcare settings might lead to low testing uptake [15, 16, 17]. Self‐collection can eliminate an unfavourable aspect of the encounter with a healthcare provider. Our findings of high acceptability were consistent with a previous study among young US transgender women [18].

The acceptance rate nearly doubled throughout the study period (from 43.9% in May to 82.8% in July). While the dramatic increase in acceptance rate may be due to word‐of‐mouth and suggestions between transgender peers in the community, we hypothesized that the biggest factor was the COVID‐19 pandemic. Because we implemented self‐collection into our services during the early stage of the COVID‐19 pandemic in our country, the increase in acceptance rate might be in part due to growing emphasis on physical distancing and the lack of provider‐collected services in our facility, which was discontinued due to the growing concern over COVID‐19 pandemic.

Based mainly on MSM studies, the CDC recommended that NAAT from self‐collected swabs is a reasonable alternative to provider‐collected rectal swabs for CT/NG testing [19]. However, there are currently no U.S. Food and Drug Administration (FDA)‐approved testing methods specifically for transgender women as well as for neovaginal specimens. Although our purpose of performance evaluation was discontinued early due to the COVID‐19 pandemic, the results were promising among eight participants. Considering that self‐collection was highly acceptable and satisfied the transgender women and showed promising performance compared to the traditional provider collection, we recommend implementing self‐collection to the STI services, particularly during the COVID‐19 pandemic where physical distancing is the new normal.

Certain limitations need to be considered. Participants who refused to perform self‐collection were unable to receive provider collection due to the COVID‐19 pandemic. Therefore, we could not compare the prevalence of CT/NG infections between the two methods. Moreover, we did not explore the reasons for the refusal. The questionnaire used to assess acceptability and satisfaction towards self‐collection did not specifically explore each anatomical site but rather the overall experience. There were substantial missing demographic data among our participants, which might lead to missing associated factors with self‐sampling acceptance. Although the performance of self‐collection was promising, we cannot conclude with great confidence due to the early discontinuation of participants enrolled in the performance evaluation between the two methods. Lastly, all self‐collection instructions in our study were in Thai. This may limit the generalizability to apply our instructions to other regions. We were able to translate the diagram for the readers. However, we made a new version of the video instruction in English after the study period to aid other regions in advocating self‐collection services for transgender women (Supporting file S3 and at https://www.youtube.com/watch?v=U6oYaDuhVmQ).

## 5 CONCLUSIONS

We demonstrated high acceptability and satisfaction towards self‐collection for CT/NG testing among Thai transgender women. The performance was promising compared to provider collection. Our results should pave the way for integrating self‐collection to the STI services, particularly during the COVID‐19 pandemic where physical distancing is the new normal. A larger study is warranted to determine the performance of self‐collection for CT/NG testing in each anatomical compartment and confirm the performance between self‐collection and provider collection.

## COMPETING INTERESTS

The authors declare that they have no competing interests.

## AUTHORS’ CONTRIBUTIONS

A.H. interpreted the data and drafted the manuscript. A.H. and C.T. performed statistical analysis. R.J. and L.H. coordinated the study and oversaw data management. T.A., P.C., K.S., A.C., J.K., J.R., P.G. and P.S. recruited, enrolled, collected data and provided technical inputs for participants. N.Te. oversaw participants. N.Th. performed laboratory testing. M.A. and S.M. approved the study initiation. R.R. and N.P. participated in study design. N.P. initiated the concept for the study. All authors critically reviewed and approved the final draft of manuscript.

## FUNDING

This work was supported by LINKAGES Project (AID‐OAA‐A‐14‐00045), which is led by FHI 360. This work was made possible by the United States Agency for International Development (USAID) and the United States President's Emergency Plan for AIDS Relief (PEPFAR) through the Linkages across the Continuum of HIV Services for Key Populations Affected by HIV (LINKAGES) project.

## DISCLAIMER

The content does not necessarily reflect the views of USAID, PEPFAR or the US Government.

## Supporting information

SUPPORTING INFORMATION

SUPPORTING INFORMATION
