# Supplementary material for: Acceptability and satisfaction towards self‐collection for chlamydia and gonorrhoea testing among transgender women in Tangerine Clinic, Thailand: shifting towards the new normal
Source: J Int AIDS Soc. 2021 Sep 8;24(9):e25801. doi: 10.1002/jia2.25801 (PMC8425782; doi:10.1002/jia2.25801)
Supplement: Supplementary file 1 — SUPPORTING INFORMATION [file JIA2-24-e25801-s001.docx]

**Supplementary File 2.** Univariate and multivariate logistic regression of characteristics associated with accepting self-collection

| **Characteristics** | **Univariate model** | | | **Multivariate model** | | |
| --- | --- | --- | --- | --- | --- | --- |
|  | **OR** | **95%CI** | **p-value** | **aOR** | **95%CI** | **p-value** |
| **Age** >25 years | 1.05 | 0.99-1.1 | 0.08 | 1.05 | 1-1.11 | 0.08 |
| **Highest education** |  |  |  |  |  |  |
| Secondary school or less | Ref | | |  |  |  |
| Vocational study | 0.97 | 0.39-2.4 | 0.94 |  |  |  |
| Bachelor degree or higher | 1.09 | 0.56-2.13 | 0.81 |  |  |  |
| **Occupation** |  |  |  |  |  |  |
| Unemployed | Ref | | |  |  |  |
| Student | 0.59 | 0.15-2.26 | 0.44 |  |  |  |
| Employed and non-sex work | 0.86 | 0.27-2.76 | 0.80 |  |  |  |
| Employed and sex work | 1.18 | 0.37-3.81 | 0.78 |  |  |  |
| **Income^1^** |  |  |  |  |  |  |
| <20000 THB/month | Ref | | |  |  |  |
| ≥20000 THB/month | 1.03 | 0.54-1.97 | 0.93 |  |  |  |
| **Marital status: single** | 1.92 | 0.12-31.11 | 0.65 |  |  |  |
| **Number of sexual partners in the past 6 months** |  |  |  |  |  |  |
| Single partner | Ref | | | Ref | | |
| Multiple partners | 1.72 | 0.34-8.79 | 0.52 | 1.42 | 0.27-7.46 | 0.68 |
| Refuse to answer | 1.94 | 0.83-4.53 | 0.13 | 2.14 | 0.9-5.06 | 0.08 |

Abbreviations: THB, Thai Baht

^1^ 20000 THB = ~ 612 USD (conversion rate 32.7 THB = 1 USD)

Multivariate logistic regression was based on covariates associated with outcomes in univariate regression with p-value of <0.15.
